# The Influence of Annealing on the Optical Properties and Microstructure Recrystallization of the TiO_2_ Layers Produced by Means of the E-BEAM Technique

**DOI:** 10.3390/ma14195863

**Published:** 2021-10-07

**Authors:** Katarzyna Jurek, Robert Szczesny, Marek Trzcinski, Arkadiusz Ciesielski, Jolanta Borysiuk, Lukasz Skowronski

**Affiliations:** 1 Deparrment of General and Inorganic Chemistry, Faculty of Chemical Technology and Engineering, Bydgoszcz University of Science and Technology, Seminaryjna 3, 85-326 Bydgoszcz, Poland; katarzyna.jurek@pbs.edu.pl; 2 Department of Analytical Chemistry and Applied Spectroscopy, Faculty of Chemistry, Nicolaus Copernicus University in Torun, Gagarina 7, 87-100 Torun, Poland; roszcz@umk.pl; 3 Institute of Mathematics and Physics, Faculty of Chemical Technology and Engineering, Bydgoszcz University of Science and Technology, Kaliskiego 7, 85-796 Bydgoszcz, Poland; marek.trzcinski@pbs.edu.pl; 4 Division of Solid State Physics, Institute of Experimental Physics, Faculty of Physics, University of Warsaw, Pasteura 5, 02−093 Warsaw, Poland; arkadiusz.ciesielski@fuw.edu.pl

**Keywords:** titanium dioxide, e-BEAM, phase transformation, microstructure, optical constants, annealing

## Abstract

Titanium dioxide films, about 200 nm in thickness, were deposited using the e-BEAM technique at room temperature and at 227 °C (500K) and then annealed in UHV conditions (as well as in the presence of oxygen (at 850 °C). The fabricated dielectric films were examined using X-ray powder diffraction, Raman spectroscopy, X-ray photoelectron spectroscopy, atomic force microscopy, scanning electron microscopy, transmission electron microscopy, and spectroscopic ellipsometry. The applied experimental techniques allowed us to characterize the phase composition and the phase transformation of the fabricated TiO_2_ coatings. The films produced at room temperature are amorphous but after annealing consist of anatase crystallites. The layers fabricated at 227 °C contain both anatase and rutile phases. In this case the anatase crystallites are accumulated near the substrate interface whilst the rutile crystallites were formed closer to the surface of the TiO_2_ film. It should be emphasized that these two phases of TiO_2_ are distinctly separated from each other.

## 1. Introduction

Titanium oxide is a widely used functional material with many applications (from bulk materials as a pigment, ceramics technology and the catalysis of nanocrystalline powder to thin layered technology). In recent years, nanocrystalline titanium oxide was developed for solar energy [1,2,3], used in biomedical and photocatalytic applications [4,5,6,7,8,9], and used as an optical [10], self-cleaning, hydrophobic [1,11,12,13] and decorative coating [14,15,16,17]. Miszczak and Pietrzyk [11] found that the transformation from anatase to the rutile phase within thin film coatings occurs in a higher temperature regime than in the case of bulk TiO_2_. They also found that the transformation temperature strongly depends on the coated substrates. The titania deposited on silicon have an anatase structure which is more stable, and therefore the phase changes occur at higher temperatures [7]. The thermodynamic factors of nanocrystalline anatase to rutile transformation were studied by Zhang and Banfield [13,18]. They proved that the smaller size of particles stabilized the anatase phase, and so the critical size was estimated to be 15 nm. Theoretical thermodynamic studies confirm that rutile has a higher surface energy than the anatase phase. The polymorph transition of titanium oxide for the wet and dry conditions of the synthesis pathway were investigated by Zhang et al. [13,18,19]. In dry conditions, the anatase phase stabilises at a particle size below 11 nm. For the particles between 11 and 35 nm in size, the brookite phase is more stable; a particle size higher than 35 nm increases the stability of rutile structure [8,9,19].

Titanium oxide is well known as one of the three important polymorphs from the metastable anatase and brookite to stabilize rutile [20]. Recently, another eight polymorphs of nanocrystalline titania were reported [13,21]. Brookite is a rare titania mineral, but recently it was investigated due to its photocatalytic efficiency (just as with anatase) [7]. The transformation from anatase to rutile is key to its applications. Nanocrystalline titania have been modified and developed [22,23] for more than a decade. The method of synthesis plays a significant role in the transformation of crystalline phases [12,24,25,26,27,28,29]. The low-pressure phases exhibit an octahedral structure, and in high-pressure phases the structure changes from octahedral to cubic. These changes are a consequence of the coordination number of titania changing from six to nine. The stability of nanocrystalline phases decreases with the number of polyhedra per unit cell volume [13,30]. The metastable anatase to rutile transformation strongly depends on oxygen defect levels [31].

Hydrothermal nanocrystalline titania were mostly investigated in the last decade [4,5,6,32,33]. Their application in modern technologies is key to modifying the properties and reactivity of TiO_2_ at the nanoscale [23]. One of the most significant aspects is understanding the relationship between structure and properties as well as the influence of thermodynamic parameters such as temperature and pressure, the synthesis pathway, and the environment on the final structure of the film. Therefore, the titania thin films have to be fabricated using a lot of techniques (e.g., ALD [17], CVD [21,28], sol-gel [8,11,21,32], magnetron sputtering [14,15,16,17,34] and e-BEAM [10,31], commonly combined with annealing after the deposition [18,19,20,21,22]). The different growing conditions of TiO_2_ leads to varying properties of these coatings and thus to various applications. In this research, the titanium dioxide films were fabricated by the electron beam physical vapor deposition (e-BEAM PVD) technique, which is a time- and cost-effective method commonly used to produce both metallic [35,36,37] and oxide coatings [38,39,40,41].

In this study, the morphology and optical properties of the titanium dioxide films produced using a e-BEAM technique were investigated by means of X-ray powder diffraction (XRD), Raman spectroscopy (Raman), X-ray photoelectron spectroscopy (XPS), atomic force microscopy (AFM), scanning electron microscopy (SEM), transmission electron microscopy (TEM), and spectroscopic ellipsometry (SE). These experimental techniques allowed us not only to characterize the phase composition and phase transformation (due to annealing at ultra-high vacuum conditions (UHV) and in oxygen) of the produced TiO_2_ films but, most of all, to show the distribution of particular polymorphs of titania inside the layer. Moreover, we have found that the annealing leads to the significant transformations in the upper part of the TiO_2_ layer.

## 2. Materials and Methods

### 2.1. Sample preparation

The titanium dioxide films with a nominal thickness of 200 nm were fabricated by means of the PVD75 e-BEAM evaporation system from Lesker (St. Leonards-on-Sea, UK). The oxide layers have been deposited from 3.5 N TiO_2_ pieces from a tungsten crucible onto polished Si substrates (100) with the native oxide. Prior to the deposition process, the substrates were mechanically cleaned with an Ar gas flow. The crucible-substrate distance was set to be 40 cm. The base pressure was 2 × 10^-^^5^ Torr. The deposition rate and total film thickness were monitored by two quartz microbalances inside the deposition chamber. The nominal deposition rate was 0.3 Å/s. 

Two sets of five samples have been fabricated. In the first series of samples (S1), the TiO_2_ layer was grown at room temperature (RT). The second set of specimens (S2) was fabricated at 227 °C. After the deposition, the samples were annealed for two or twelve hours at 850 °C. The annealing was then performed in a UHV preparation chamber (base pressure ≤ 5 × 10^-^^10^ mbar). Moreover, the set of specimens was heated in oxygen (1·10^−5^ mbar) at 850°C, the purity of the oxygen was 4.5 (99.995%). All of the prepared samples are summarized in Table 1.

### 2.2. Sample characterization

The surface topography of the prepared TiO_2_ films were examined using an Atomic Force Microscope (AFM) Innova (Bruker, Billerica, MA, USA) with the standard Si tips for a tapping mode and scanning electron microscopy (SEM). The SEM measurements were performed with a Quanta 3D FEG (FEI, Hillsboro, OR, USA) (EHT = 30 kV) instrument. 

Specimens for cross-sectional transmission electron microscopy (TEM) measurements were prepared using a standard method of mechanical pre-thinning followed by Ar ion milling. TEM characterization was performed using Titan 80–300 and Talos F200X microscopes from FEI, operating at 300 keV and 200 keV, respectively. The measurements were performed both in bright field (BF) and in high-angle annular dark field (HAADF) modes.

XRD patterns were registered using the Phillips X’Pert (Malvern Panalytical Ltd., Malvern, UK) device with Cu Kα radiation (λ = 1.5418 Å) equipped with the X’Celerator Scientific (Malvern Panalytical Ltd., Malvern, UK) detector. These measurements were performed in the range 2θ from 20° to 70°.

Raman spectra were recorded using Bruker SENTERRA confocal microscope (Bruker Optik, Ettlingen, Germany) in the spectral range of 90–1600 cm^−1^. The Raman scattering was excited using the near-infrared laser operating at 785 nm.

The X-ray photoelectron spectroscopy (XPS) analysis was carried out under the UHV conditions (base pressure ≤ 2 × 10^-^^10^ mbar). The Al K_α_ source (1486.6 eV) at 55 degrees with respect to the normal of the sample was used as an incident radiation. The energy of photoelectrons was analyzed by the VG-Scienta R3000 (Uppsala, Sweden) spectrometer (the energy step was set at ΔE = 0.1 eV. The CasaXPS software (version 2.3.16, Casa Software Ltd., Teignmouth, UK) was used for quantitative analysis (the Gauss–Lorentz shapes were used to fit the experimental data).

Ellipsometric azimuths Ψ and Δ were collected for three angles of incidence (65°, 70° and 75°) in the NIR-vis-UV spectral range (193–2000 nm; 0.6–6.5 eV) using the V-VASE (from J.A.Woollam Co., Inc.; Lincoln, NE, USA.) instrument. The thickness of TiO_2_ films as well as their optical constants were established using WVASE32 software (J.A.Woollam Co., Inc.; Lincoln, NE, USA.).

## 3. Results and Discussion

The obtained titanium dioxide films were characterized using AFM, SEM, Raman, XRD, XPS, and spectroscopic ellipsometry (SE) measurements. In this section the results related to the composition of the TiO_2_ layers are presented. Next, the findings associated with the topography of the layer and thickness of the prepared dielectric films are shown. Finally, the optical properties of the fabricated titanium dioxide layers are discussed. We would like to emphasize that the optical model of a sample used during the study of ellipsometric measurements directly results from the analysis of the morphology of the thin films.

**Figure 1:**
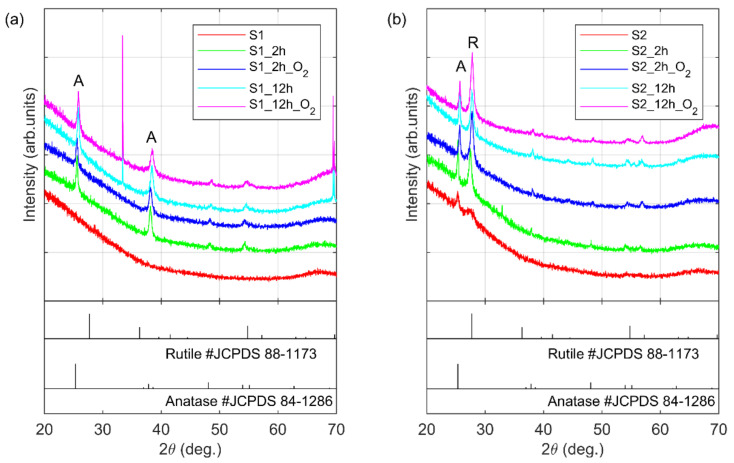
XRD patterns recorded for samples from the (**a**) S1 and (**b**) S2 series of samples.

X-ray diffraction patterns of thin layers of TiO_2_ registered for S1 and S2 series of samples are shown in Figure 1. The nanocrystalline titania films deposited in two temperatures (RT and 227 °C) exhibit different structures. In Figure 1a no peaks are observed for the sample deposited at room temperatures (S1). This corresponds to amorphous TiO_2_. For the sample deposited at a higher temperature (see Figure 1b, the S2 specimen) two peaks are observed. The signal at an angle of 2θ≈25° corresponds to anatase (101), while the one at 2θ≈27° can be attributed to the rutile (110) polymorph of TiO_2_. It should be noted that the peak at 2θ≈27° exhibits low intensity. For the S2 specimen, an increase of crystallinity was observed. After 2h of annealing of the S1 sample in 850 °C in UHV conditions two signals at angles of 25° and 38° corresponding to anatase (101 and 004) have been detected (the S1_2h sample). Similar diffraction structure was obtained for the sample annealed by 12h (S1_12h). For samples annealed in the UHV chamber in oxygen (S1_2h_O2 and S1_12h_O2), two peaks at the angles ~25^o^ and ~38^o^ have been detected. However, their intensity is lower than of those recorded for samples obtained by annealing without oxygen. At angles ~48° and ~53° 2θ, two signals with very low intensity correspond to anatase structures are detected. Very intense signal at angle 33° comes from silicon wafer substrates Si (100). For series S2, after annealing samples in UHV condition both anatase (101) and rutile (110) signals have increased in intensity. The other signals in these diffractogram exhibit very low intensity and correspond to the anatase structure. In the presence of oxygen in the UHV chamber for the S2 series of samples, an increased intensity of the rutile (110) signal at an angle of 27° was found. The thin layers of TiO_2_ deposited on the silica substrate by the PVD e-beam method in two different temperatures are varied structurally. As Miszczak and Pietrzyk [11] have shown, the coated titanium oxide polymorph transformation requires more energy than the same process in a powder sample. Therefore, the transformation phase in the thin layer occurs at a higher temperature. The impurities of the material as well as the type of substrate used during the deposition impact this process. Many studies show that dopant and substrate are inhibiting transition phase structures [11,21,22]. It is well known that the silica surface is the most inhibiting agent of anatase to the rutile conversion process. The inhibiting character is manifested by a higher temperature of transition of the metastable anatase to the rutile structure. Therefore, no anatase-to-rutile phase transition is observed for the S1 set of samples, even in the case of annealing them in 850 °C for 12 hours. However, for the S2 set of samples, where a small number of rutile crystals are present in an as-fabricated sample, the rutile-associated signals rise in intensity with annealing. This suggests that the already present rutile crystals act as recrystallization seeds during annealing, lowering the activation energy for anatase-to-rutile phase transition. Therefore, for the S2 set of samples, some anatase crystals undergo the phase transition during annealing. It should be noted that the above-mentioned description is related to transformations of TiO_2_. However, the high-baseline of XRD patterns at 2θ = 20–40° can be attributed to amorphous titania, which still exists in the film.

The average size of TiO_2_ crystallites was estimated using the Scherrer equation:

(1) $$\langle D\rangle =\frac{0.94\lambda }{\beta cos\theta }$$

In Equation (1) *<D>* is the average crystallite size, β is the line broadening, θ is the Bragg angle, and λ is the X-ray wavelength. In Table 2 the decreasing of crystallite size of anatase (101) was observed for the series S1 samples annealed in the presence of oxygen for both durations. For the S2 samples annealed for 2h and 12h in UHV conditions, anatase (101) crystalline size increased significantly compared to the sample in RT. For samples annealed in the presence of oxygen, only a slight increase of crystalline size (+4 nm) was detected in the case of 12h annealing an insignificant increase (+1 nm) was detected for 2h of annealing. For anatase (004) the average size is almost the same for all S1 samples. In the S2 series the rutile crystallite size significantly increased after annealing the samples with or without the presence of oxygen. These results indicate that neither the time of annealing nor the presence of oxygen during the annealing have no significant impact on the phase transformation process. 

Some works reported that the transformation anatase to rutile occurs with the increasing of the crystallite size [8,13,19,21,22]. Only temperature deposition by e-beam PVD significantly changes the structure of the thin TiO_2_ layer. A lower temperature of deposition stabilized the anatase phase and increased the transformation temperature. Hanaor and Sorrell [21] have utilized the data from the literature to summarize and report the potential effect of various dopants on anatase-to-rutile phase transformation. Silicon has been reported as a type of dopant which inhibits the process. The Si atoms are stabilizing the anatase phase, as they are incorporated into the crystal lattice, forming the Ti^4+^ interstitial defect, which leads to distortion of the anatase lattice. This leads to increased activation energy of the lattice contraction associated with the anatase-to-rutile transformation. The authors of [21] also reported that the undissolved SiO_2_ reduced the anatase interparticle contact distance, thus increasing the number of heterogeneous nucleation sites. Based on these considerations, the influence of substrate on the phase transformation process was reported by Miszczak and Pietrzyk [11]. The increasing of deposition temperature can be assigned to both the anatase and rutile phases. 

**Figure 2:**
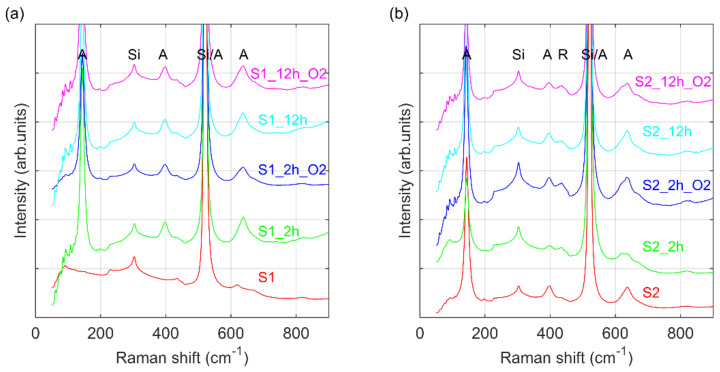
Raman spectra for: (**a**) S1 and (**b**) S2 series of samples

The Raman spectrum of thin layer S1 (see Figure 2a) shows peaks at 303 cm^−1^, 514 cm^−1^, and a broad signal from 940 cm^−1^ to 980 cm^−1^ from the silicon wafer. Thus, both Raman and XRD analysis confirm the amorphous form of titania. For samples S1 annealed in both conditions, with or without the presence of oxygen, three new peaks are observed at 142, 395, and 636 cm^−1^ corresponding to the E_g_(1), B_1g_(1), and E_g_(3) modes of anatase. The very intense peak at 514 cm^−1^ corresponds to both anatase A_1g_&B_1g_(2) and silicon wafer modes. The E_g_ mode is associated with symmetric stretching, and B_1g_ belongs to symmetric bending vibrations of O-Ti-O group. When the temperature of deposition is increased for the S2 samples, a new signal at 437 cm^−1^ emerges, which can be attributed to asymmetric stretching of O-Ti-O bonding of rutile (110) plane [5,6,9,25]. 

Both XRD and Raman measurements confirm an increase in the crystallinity of the fabricated TiO_2_ layers. The titania films grown at RT are amorphous. The annealing process leads to formation of anatase grains (the S1 series of samples). In the TiO_2_ layers deposited at higher temperature both rutile and anatase crystallites are present and thermal treatment causes an increase in both polymorphs of titania (the S2 series of samples).

**Figure 3:**
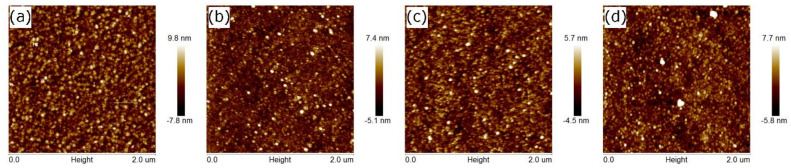
AFM images of (**a**) S1; (**b**) S2; (**c**) S1_O2_12h; and (**d**) S2_O2_12h samples.

**Figure 4:**
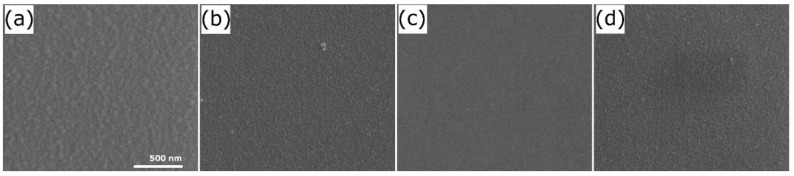
SEM images of (**a**) S1; (**b**) S2; (**c**) S1_O2_12h; and (**d**) S2_O2_12h samples.

The surface topography of the TiO_2_ films examined by AFM and SEM techniques is presented in Figure 3 and Figure 4. All of the samples exhibit a uniform nanogranular structure with the lateral grain size of 30–50 nm (see Figure 3). It should be noted that one separate grain demonstrates extended surface. To describe the smoothness of the obtained TiO_2_ films the roughness parameters have been calculated. Quantities R_a_ (the arithmetical mean deviation of the assessed profile):

(2) $$R_{a}=\frac{1}{N}\overset{N}{\underset{j=1}{\sum }}\left|Z_{j}\right|$$

and *R_q_* (the root mean squared roughness):

(3) $$R_{q}=\sqrt{\frac{1}{N}\overset{N}{\underset{j=1}{\sum }}Z_{j}^{2}}$$

were calculated using the NanoScope Analysis software (version 1.40). In Equations (2) and (3) Z_j_ is the current surface height value and N is the number of points measured. The calculated values of *R_a_* and *R_q_* are summarized in Table 2. The calculated *R_a_* values are in the range from 0.8 nm to 2.0 nm, while *R_q_* values are in the range from 1.0 to 2.6 nm. The roughness parameters do not show any dependence on the temperature during deposition or on the time of annealing. The thickness of the rough layer (d_r_; see Table 2) estimated from spectroscopic ellipsometry measurements are comparable to or lower than the roughness parameters. The thickness of the titanium dioxide layer (d_1_ + d_2_; see Table 2) for the S1 series of samples is ~220–230 nm and ~180 nm for the S2 series of samples. It means that the surface roughness constitutes about 1% of the thickness of the TiO_2_ film. 

**Figure 5:**
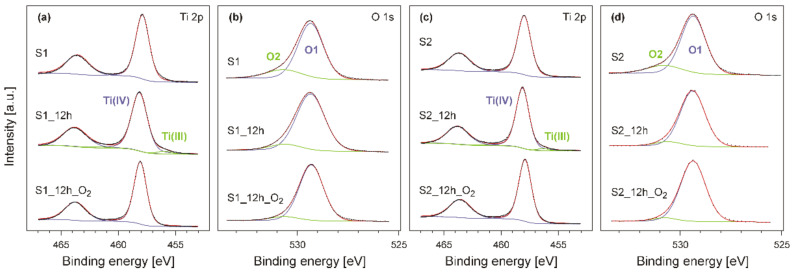
XPS spectra of titanium 2p ((**a**) and (**c**)) and oxygen 1 s ((**b**) and (**d**)) levels recorded for S1 ((**a**) and (**b**)) and S2 ((**c**) and (**d**)) series of samples

The results of XPS measurements (for the Ti 2p and O 1s regions) for the produced titanium dioxide films are presented in Figure 5. The position of the Ti 2p_3/2_ peak ranges from 457.8 eV to 458.1 eV can be attributed to the Ti^4+^ (see Figure 5a and 5c; Ti(IV)) oxidation state, thus providing evidence for the presence of titanium dioxide on the surface of the specimens [42,43]. The slight shift of the Ti^4+^ signal towards higher binding energies during the thermal treatment can be attributed to the phase transformation, and this effect was observed in the earlier investigations [34,42]. Annealing in a vacuum (without oxygen) at 850 °C leads to partial reduction of TiO_2_. As a result, titanium(III) has been formed. The concentration of the Ti(III) part was estimated to be at the level of 2–3%. The low concentration of the compound indicates that the Ti_2_O_3_ crystallites were not detected in the XRD measurements (see Figure 1). This effect was not observed for the samples annealed in oxygen; the XPS spectra recorded for Ti 2p level contain only one component. The oxygen 1s signal reveals a double structure of the peak. Its most prominent compound (O1, see Figure 5b,d) centred at about 529 eV comes from Ti-O bonds. The second component (O2) is significantly less intensive, but is clearly observable on the all XPS spectra recorded for O 1s level at about 531 eV. This signal is a result of the contamination of the surface [44] of TiO_2_. This effect is associated with the fact that the produced titanium dioxide films had contact with the atmosphere before being introduced into the UHV system.

**Figure 6:**
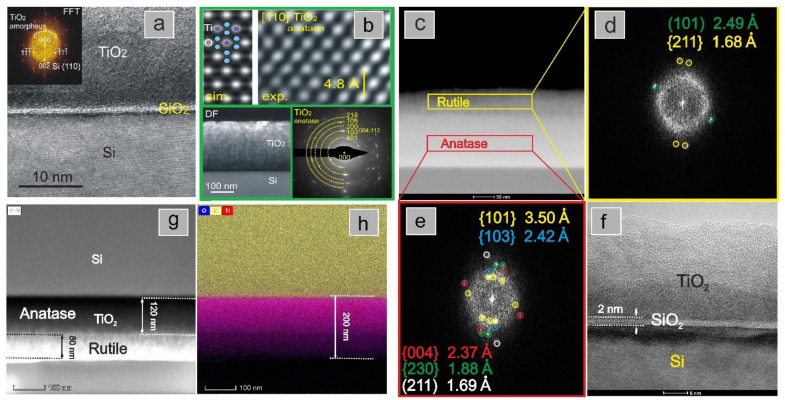
Characterisation of TiO_2_ layers(cross-sections) by transmission electron microscopy: (**a**) a TEM image and fast Fourier transform (FFT) pattern (inset) of a sample S1; (**b**) inverse fast Fourier transform (IFFT) image of sample S1_12h (top right) and atomic model of anatase with the corresponding simulated IFFT image along the axis {110} (top left), and TEM image of a layer (bottom left) with a selected area electron diffraction (SAED) pattern taken from TiO_2_ (bottom right); (**c**) TEM image of sample S2 with fast-Fourier-Transform (FFT) images taken from the marked areas of the cross-section presented in (**d**) and (**e**) with high resolution TEM image of the layer (**f**); (**g**) a TEM image of the sample S2_12h with determined thicknesses of anatase and rutile phase with the corresponding EDS analysis (**h**).

The registered TEM images as well as the fast-Fourier-transform (FFT) images (Figure 6a) of the sample S1 reveal the amorphous nature of the deposited film. The layer thickness is about 250 nm. The thermal treatment of the material (sample S1_12h) led to the formation of a crystalline layer on the Si/SiO_2_ surface. The selected area electron diffraction (SAED) pattern confirmed the presence of the anatase (Figure 6b). Moreover, the image generated from high resolution TEM inverse fast Fourier transform (IFFT) image is consistent with the simulated one (Figure 6b). Estimated for appropriate HRTEM image lattice distance of 0.23 nm matches well with the expected spacing of the anatase TiO_2_ (112) plane. In contrast to the sample deposited on cold substrates, well-defined crystalline material is observed for S2 layer. The TEM images collected by the dark field (DF) detector, which is sensitive to phase changes, indicate a separation in the TiO_2_ film (Figure 6c). The FFT analysis of HR TEM image (Figure 6f), shows that the bottom layer has more observable planes which may correspond to the structure of anatase (Figure 6e). The rutile phase does not have so many probably present planes (Figure 6d) and only a group of two strong reflections is visible for a top layer. Additionally, the energy-dispersive X-ray spectroscopy (EDS) shows a distinct boundary between the TiO_2_ and Si phases, indicating that the titanium dioxide phase does not penetrate the substrate. The estimated thickness of SiO_2_ is about 2 nm. The subsequent heating of the S2 sample material does not significantly change the phase arrangement. The determined thickness of anatase for S2_12h is about 120 nm and for rutile is 80 nm (Figure 6g). The clear separation of the silicon- and titanium-containing phases is still visible (Figure 6h). These results clearly indicate that during the fabrication of the TiO_2_ layers at elevated temperatures (S2 set of samples), the anatase phase is formed first. The effects of the type of substrate and its temperature during the deposition on nucleation and crystallisation processes have been recently investigated [45,46,47,48,49]. These studies have shown that the morphology of the deposited TiO_2_ films is strongly influenced by the diffusion of oxygen from thin titania layer into the substrate. As a consequence of oxidation and reduction processes at the interface, nonstoichiometric oxides are formed and the nucleation and crystallisation processes occur in higher temperatures than in the absence of the substrate. Moreover, the crystallisation and nucleation processes are impacted by the deposition temperature [45,46,47,48,49]. At low temperatures (below 300 °C), smooth layers of amorphous TiO_2_ were formed [48]. At higher temperatures, the nucleation centres of both the amorphous and anatase phases were formed [49].

As the layer grows, the influence of the substrate layer on the titanium and oxygen adatoms becomes weaker, allowing the rutile phase to be formed. Combining these results with results from X-ray analysis allows us to conclude that for the S2 series of samples, the anatase-to-rutile recrystallization front goes in the direction from the surface towards the substrate interface. This is consistent with previous reports, where the formation of anatase crystals within the amorphous phase of TiO_2_ was investigated [45,49]. It has been shown that the anatase seeds emerge at the surface of the TiO_2_ layer only after a critical film thickness has been achieved. In the case of [45], the critical thickness was estimated to be between 30 and 40 nm. As the film thickness increased, the anatase grains grew, acting as recrystallization seeds for the amorphous phase in their vicinity. In case of our films, a similar phenomenon is observed (however with rutile grains). The fact that rutile which only formed near the surface of the TiO_2_ film may be a result of the overall film thickness being too small for full anatase-to-rutile recrystallization.

**Figure 7:**
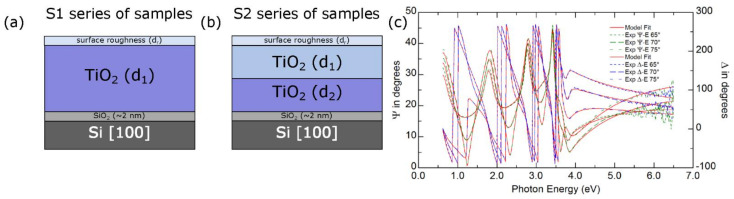
Optical models of a sample used to analyze (**a**) the S1 set of samples and (**b**) S2 series of samples. (**c**) The experimental Ψ and Δ ellipsometric azimuths measured for three angles of incidence (65°, 70°, and 75°) and their model fits for the S1 sample.

Taking into account the structure of the obtained titania films established during the analysis of TEM measurements, we propose two optical models of samples. The first one (Si\native silica\titania\rough layer\ambient) is dedicated for the S1 series of samples (see Figure 7a). In this model, the TiO_2_ film is amorphous (the S1 sample) or consists of anatase grains (the other samples in S1 series). The second model presented in Figure 6b contains two separate TiO_2_ films of thicknesses d_1_ and d_2_. Based on the TEM measurements, it can be assumed that the layers marked in Figure 7b as TiO_2_(d_1_) and TiO_2_(d_2_) can be assigned to rutile and anatase polymorphs of titanium dioxide, respectively.

Independently from the selected optical model of samples, its parameters were adjusted to minimize the mean squared error (χ^2^), which was defined as [50,51,52]:

(4) $$\chi^{2}=\frac{1}{N-P}\sum_{j}\left({\left(\Psi_{j}^{mod}-\Psi_{j}^{exp}\right)}^{2}+{\left(\Delta_{j}^{mod}-\Delta_{j}^{exp}\right)}^{2}\right).$$

In Equation (4), N and P are the total number of data points and the number of fitted model parameters, respectively, while the quantities $\Psi_{j}^{mod},\;\Psi_{j}^{exp},\Delta_{j}^{mod}\;and\;\Delta_{j}^{exp}$ are the experimental (the quantities with superscript “exp”) and calculated (the quantities with superscript “mod”) ellipsometric azimuths. An example of the fit for the S1 sample has been presented in Figure 7c. The calculated values of χ^2^ are lower than 4.4 d for all of the examined samples. 

Generally, the refractive index $\left(\tilde{n}\right)$ is a complex quantity:

(5) $$\tilde{n}=n-ik,$$

where n and k are the real part of $\tilde{n}$ and the extinction coefficient, respectively. The optical constants of Si and SiO_2_ were taken from the database of optical constants [50]. The complex refractive index of the titanium dioxide layer (or layers for the S2 set of samples; see Figure 7b) was parameterized using the sum of Pole (equivalent of the Sellmeier dispersion relation), Tauc-Lorentz, and Gaussian oscillators [50,51]:

(6) $${\tilde{n}}^{2}=\varepsilon_{\infty }+Pole\left(A_{P},E_{P}\right)+T-L\left(A_{T-L},E_{g},E_{n},Br_{T-L}\right)+\underset{j}{\sum }Gauss\left(A_{j},E_{j},Br_{j}\right).\;$$

In Equation (6), $\varepsilon_{\infty }$ is a high-frequency dielectric constant, while A_k_, E_k_, and Br_k_ are the amplitude, energy, and broadening of an oscillator. The quantity E_g_ is the band-gap energy of the Tauc-Lorentz oscillator. It should be noted that for the S2 set of samples the complex refractive index was determined independently for each layer (see Figure 7b). Mathematical formulas of particular line-shapes can be found in [50,51]. Optical constants of the roughness have been described as a Bruggeman effective medium approximation (BEMA) [50,51] with fractions of void (ambient) and dielectric (TiO_2_) kept at 0.5:

(7) $$0.5\frac{n_{v}^{2}-{\tilde{n}}_{r}^{2}}{n_{v}^{2}+2{\tilde{n}}_{r}^{2}}+0.5\frac{{\tilde{n}}_{TiO2}^{2}-{\tilde{n}}_{r}^{2}}{{\tilde{n}}_{TiO2}^{2}+2{\tilde{n}}_{r}^{2}}=0.$$

In Equation (7) n_v_ (n_v_ = 1), ${\tilde{n}}_{TiO2}^{}$ and ${\tilde{n}}_{r}^{}$ are refractive indexes of void, titanium dioxide and the rough layer, respectively.

**Figure 8:**
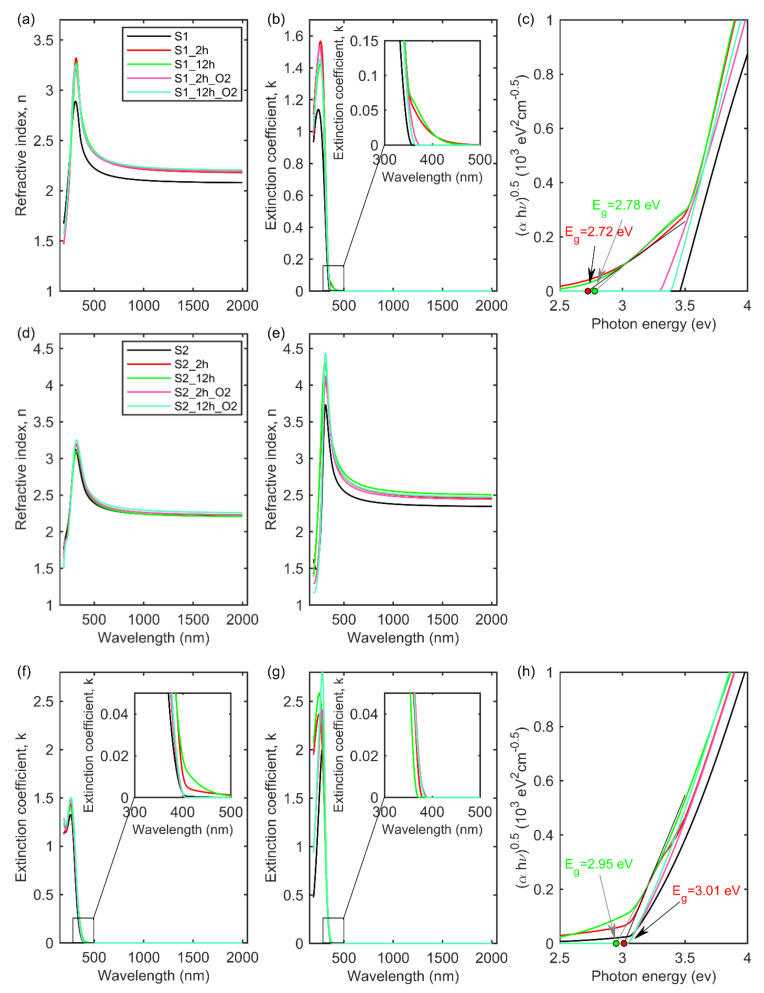
(**a**) Refractive index, (**b**) extinction coefficient, and (**c**) the Tauc plot for the S1 set of samples. (**d**) Refractive index of TiO_2_(d_1_) and (**e**) TiO_2_(d_2_) layers for the S2 set of samples (see Figure 6b). (**f**) Extinction coefficient of TiO_2_(d_1_) and (**g**) TiO_2_(d_2_) layers for the S2 series of specimens. (**h**) The Tauc plot for the S2 series of samples determined based on the k spectra presented in (**f**).

The determined optical constants of the investigated dielectric films are shown in Figure 8. The refractive index (Figure 8a) calculated for the S1 series of samples exhibits a normal dispersion relation in the vis-NIR spectral range. In the non-absorbing spectral range (k = 0; see Figure 8b) the values of n for the as-deposited TiO_2_ film are about 2.15 (amorphous film). Annealing in air or vacuum leads to increase in the value of the refractive index to about 2.25. This change is associated with the formation of the anatase polymorph of TiO_2_. This fact has been confirmed in XRD (see Figure 1) and TEM (see Figure 7) measurements. For wavelengths below 400 nm, the strong absorption band is observable (see Figure 8b). The absorption threshold for the deposited TiO_2_ (S1) and oxidized (S1_2h_O2 and S1_12h_O2) films can be estimated directly from the Tauc-Lorentz oscillator (the Eg quantity; see Table 3). However, the annealed dielectric layers (S1_2h and S1_12h) exhibit quite a high k value in the spectral range from 360 nm to 450 nm (see an inset in Figure 8b). This absorption feature is associated with the existence of Ti(III) compound in the XPS spectrum (see Figure 5a) [48]. The concentration of Ti(III) compound in the subsurface region was estimated to be 2–3%. In Equation 6, this additional absorption band is described as a gaussian oscillator. Therefore, to estimate the value of the band-gap energy, the method based on the Tauc-plot has been implemented [53]. This approach is widely used to determine the value of an absorption threshold [34,54]. The Tauc plot was implemented by plotting (αhν)^1/m^ against photon energy (hν), where m is the parameter associated with the type of transition and may take a value of 1/2, 3/2, 2, or 3 for direct allowed transition, direct forbidden transition, indirect allowed transition, and indirect forbidden transition, respectively [53]. A value of m equal to 2 was chosen for this analysis. The estimated band-gap energy for the deposited and annealed in oxygen TiO_2_ films (S1, S1_2h_O2 and S1_12h_O2 specimens) are 3.46 eV, 3.32 eV, and 3.39 eV, respectively (see Table 3). These values are slightly higher than that obtained for amorphous or anatase polymorphs of titanium dioxide [34,55,56]. These discrepancies are most probably associated with the quantum size effect. TiO_2_ crystallites this effect and can cause the blue shift of the absorption threshold up to 0.2 eV [57]. Values of Eg determined for the annealed in vacuum dielectric films are 3.47 eV and 3.50 eV for the S1_2h and S1_12h samples, respectively. It should be noted that these values cannot be ascribed to the band-gap energy of a whole film due to the existence of the additional absorption band for wavelengths longer than 360 nm. Therefore, these values should be treated as parameters in mathematical formulas used to parameterize optical constants of a film or can be ascribed to the stoichiometric TiO_2_ (Ti(IV) component in XPS measurements; see Figure 5a). In this case, the band-gap energy of the produced layer is 2.72 eV and 2.78 eV for S1_2h and S1_12h specimens, respectively.

The films from the S2 series of samples are composed of two separated layers containing anatase (bottom layer) and rutile (upper layer) crystallites (see Figure 6). The thickness is in the range from 75 nm to 78 nm and from 99 nm to 115 nm for rutile and anatase parts of the TiO_2_ coating, respectively (see Table 2). This result is in a good agreement with the thicknesses obtained from TEM studies (see Figure 6). In the non- absorbing spectral range, a value of the refractive index is about 2.50-2.55 for the anatase film (see Figure 8e), while the band-gap energy is 3.18–3.35 eV (see Figure 8g and Table 3). The obtained results proved good quality of the close-packed anatase film [58]. A different situation can be observed for the rutile film. In the NIR-vis spectral range a value of the refractive index is only ~2.4, which is lower than that obtained for anatase film in this study (see Figure 8b,e) and lower than that obtained for rutile close-packed films [57]. This result can be explained as an effect of nanoporosity of the obtained TiO_2_ films [34,57,58]. The values of band-gap energy for all rutile films are 3.04–3.05 eV. This result is in a good agreement with a value of Eg reported earlier for rutile polymorph of TiO_2_ [34,56]. As in the case with the S1 series of samples annealed in a vacuum, the thin oxide layers the additional absorption band (related to the Ti(III) compound found the XPS measurements) can be visible, which causes the blue shift of the band-gap energy to 3.01 eV and 2.95 eV for S2_2h and S2_12h samples, respectively.

## 4. Conclusions

TiO_2_ layers about 200 nm thick were prepared using the e-BEAM PVD technique. Titanium dioxide was deposited on a Si substrate at room temperature and at 227 °C (500K) and then annealed in both UHV alone and UHV with oxygen conditions at 850 °C. The TiO_2_ coatings were investigated using XRD, Raman spectroscopy, XPS, AFM, SEM, TEM, and SE techniques. We have shown that the annealing of an amorphous titania layer leads to the formation of anatase crystallites with two crystallographic orientations. The films deposited at 227 °C contain both anatase and rutile phases, wherein the anatase crystallites are a few times as large as the rutile ones. Annealing leads to the growth of rutile crystallites, since the already present rutile grains act as recrystallization seeds and allow for the anatase-to-rutile phase transition. Based on the performed investigations, we can conclude that 2h of annealing has to be sufficient to complete the transformation in the TiO_2_ film. The annealing by 12h was carried out to be sure that the transformation process has been completed in the given conditions. It does not mean that the whole amorphous TiO_2_ has been transformed into the crystallite phase of titania. Rather, it means that the equilibrium state has to be achieved and that a longer time of thermal treatment does not lead to further phase transformations.

It should be noted that both polymorphs of TiO_2_ are distinctly separated from each other. The anatase crystallites are formed at the interface of the Si substrate, while the rutile crystallites near the surface of the titanium dioxide layer. Thus, it can be concluded that the anatase-to-rutile recrystallization front goes from the surface towards the substrate interface. Generally, annealing in oxygen leads to the growth of crystallites, while annealing in a vacuum causes partial decomposition of TiO_2_. The band-gap energy for the produced anatase films is close to or slightly larger than that obtained for the close-packed anatase TiO_2_ films. The absorption threshold for the rutile layer lies at the energy reported for rutile. Simultaneously, it should be emphasized that the value of band-gap energy found for the layers annealed in a vacuum exhibit a red-shift of their positions.

## Figures and Tables

**Table 1:** A list of the prepared samples (T_s_, the temperature of substrate during deposition; t, time of annealing; T_a_, the temperature of annealing; O_2_, existence of oxygen during annealing).

No.	Sample	T_s_	T	T_a_	p_O2_
		(°C)	(h)	(°C)	(mbar)
1.	S1	RT ^1^	-	-	-
2.	S1_2h	RT ^1^	2	850	-
3.	S1_2h_O2	RT ^1^	2	850	10^-5^
4.	S1_12h	RT ^1^	12	850	-
5.	S1_12h_O2	RT ^1^	12	850	10^-5^
6.	S2	227	-	-	-
7.	S2_2h	227	2	850	-
8.	S2_2h_O2	227	2	850	10^-5^
9.	S2_12h	227	12	850	-
10.	S2_12h_O2	227	12	850	10^-5^

^1^ RT, room temperature.

**Table 2:** The thickness of TiO_2_ layers (d_1_ and d_2_) and the rough layer (d_r_), roughness parameters (R_a_ and R_q_), and the estimated crystallite size (*<D>*).

No.	Sample	d_r_	d_1_	d_2_	R_a_	R_q_	*<D>*	*<D>*
		(nm)	(nm)	(nm)	(nm)	(nm)	(nm)	(nm)
1.	S1	2.3 ± 0.2	234 ± 1	-	2.0	2.6	-	-
2.	S1_2h	0.6 ± 0.2	223 ± 1	-	0.8	1.0	32 ± 3 ^1^	22 ± 2 ^2^
3.	S1_2h_O2	0.8 ± 0.2	231 ± 1	-	1.4	1.7	25 ± 3 ^1^	21 ± 2 ^2^
4.	S1_12h	0.8 ± 0.2	224 ± 1	-	1.2	1.5	32 ± 3 ^1^	21 ± 2 ^2^
5.	S1_12h_O2	0.5 ± 0.2	222 ± 1	-	1.1	1.4	25 ± 3 ^1^	20 ± 2 ^2^
6.	S2	2.9 ± 0.2	78 ± 1	115 ± 1	1.3	1.7	29 ± 3 ^1^	5 ± 4 ^3^
7.	S2_2h	1.9 ± 0.3	77 ± 1	101 ± 1	1.7	1.5	41 ± 4 ^1^	19 ± 2 ^3^
8.	S2_2h_O2	2.8 ± 0.2	75 ± 1	108 ± 1	1.4	2.0	30 ± 3 ^1^	19 ± 2 ^3^
9.	S2_12h	1.9 ± 0.2	78 ± 1	99 ± 1	1.2	1.6	41 ± 4 ^1^	20 ± 2 ^3^
10.	S2_12h_O2	2.2 ± 0.2	73 ± 1	105 ± 1	1.1	1.4	33 ± 3 ^1^	17 ± 2 ^3^

^1^ Anatase (hkl) = (101). ^2^ Anatase (hkl) = (004). ^3^ Rutile (hkl) = (110).

**Table 3:** Values of the band-gap energy determined from the Tauc-Lorentz oscillator (Eg_1_ and Eg_2_) and based on the Tauc-plot method (Eg_T-L_)

No.	Sample	Eg_1_		Eg_2_		Eg_T-L_	
		(eV)	(nm)	(eV)	(nm)	(eV)	(nm)
1.	S1	3.46 ± 0.01	358 ± 1	-	-	-	-
2.	S1_2h	3.47 ± 0.01	357 ± 1	-	-	2.72 ± 0.05	456 ± 9
3.	S1_2h_O2	3.31 ± 0.01	375 ± 1	-	-	-	-
4.	S1_12h	3.50 ± 0.01	354 ± 1	-	-	2.78 ± 0.05	446 ± 8
5.	S1_12h_O2	3.39 ± 0.01	366 ± 1	-	-	-	-
6.	S2	3.18 ± 0.03	390 ± 4	3.05 ± 0.05	407 ± 7	-	-
7.	S2_2h	3.27 ± 0.03	379 ± 4	3.05 ± 0.07	407 ± 10	3.01 ± 0.05	412 ± 7
8.	S2_2h_O2	3.20 ± 0.04	387 ± 5	3.05 ± 0.06	407 ± 8	-	-
9.	S2_12h	3.35 ± 0.02	370 ± 3	3.04 ± 0.04	408 ± 6	2.95 ± 0.05	420 ± 8
10.	S2_12h_O2	3.20 ± 0.03	387 ± 4	3.05 ± 0.04	407 ± 6	-	-

## Data Availability

Data are available from the corresponding author.

## References

[1] Elsaeedy H., Qasem A., Yakout H., Mahmoud M. (2021). The pivotal role of TiO_2_ layer thickness in optimizing the performance of TiO_2_/P-Si solar cell. J. Alloy. Compd..

[2] Cardoso B.N., Kohlrausch E.C., Laranjo M.T., Benvenutti E., Balzaretti N., Arenas L., Santos M.J.L., Costa T.M.H. (2019). Tuning anatase-rutile phase transition temperature: TiO_2_/SiO_2_ nanoparticles applied in dye-sensitized solar cells. Int. J. Photoenergy.

[3] Gouma P.I., Mills M.J. (2001). Anatase-to-rutile transformation in titania powders. J. Am. Ceram. Soc..

[4] Wetchakun N., Incessungvorn B., Wetchakun K., Phanichphant S. (2012). Influence of calcination temperature on anatase to rutile phase transformation in TiO_2_ nanoparticles synthesized by the modified sol-gel method. Mater. Lett..

[5] Hong Q.-M., Wang S.-Y., An D.-L., Li H.-Y., Zhou J.-M., Deng Y.-F., Zhou Z.-H. (2019). Transformations of dimeric and tetrameric glycolato peroxotitanates and their thermal decompositions for the preparations of anatase and rutile oxides. J. Solid State Chem..

[6] Loan T.T., Huong V.H., Huyen N.T., Van Quyet L., Bang N.A., Long N.N. (2021). Anatase to rutile phase transformation of iron-doped titanium dioxide nanoparticles: The role of iron content. Opt. Mater..

[7] Badovinac I.J., Peter R., Omerzu A., Salamon K., Šarić I., Samaržija A., Perčić M., Piltaver I.K., Ambrožić G., Petravić M. (2020). Grain size effect on photocatalytic activity of TiO_2_ thin films grown by atomic layer deposition. Thin Solid Film..

[8] Anitha B., Khadar M.A. (2020). Anatase-rutile phase transformation and photocatalysis in peroxide gel route prepared TiO_2_ nanocrystals: Role of defect states. Solid State Sci..

[9] Bassi A.L., Cattaneo D., Russo V., Bottani C.E., Barborini E., Mazza T., Piseri P., Milani P., Ernst F.O., Wegner K. (2005). Raman spectroscopy characterization of titania nanoparticles produced by flame pyrolysis: The influence of size and stoichiometry. J. Appl. Phys..

[10] Sangani L.D.V., Mohiddon A., Rajaram G., Krishna M.G. (2019). Optical confinement in TiO_2_ waveguides fabricated by resist free electron beam lithography. Opt. Laser Technol..

[11] Miszczak S., Pietrzyk B. (2015). Anatase-rutile transformation of TiO_2_ sol-gel coatings deposited on different substrates. Ceram. Int..

[12] Hsiang H.-I., Lin S.-C. (2008). Effects of aging on nanocrystalline anatase-to-rutile phase transformation kinetics. Ceram. Int..

[13] Zhang H., Banfield J.F. (2014). Structural characteristics and mechanical and thermodynamic properties of nanocrystalline TiO_2_. Chem. Rev..

[14] Skowronski L., Chodun R., Zdunek K. (2020). TiO_2_—based decorative interference coatings produced at industrial conditions. Thin Solid Film..

[15] Skowronski L., Wachowiak A., Grabowski A. (2016). Characterization of optical and microstructural properties of semitransparent TiO_2_/Ti/glass interference decorative coatings. Appl. Surf. Sci..

[16] Skowronski L., Wachowiak A., Wachowiak W. (2017). Optical and microstructural properties of decorative Al/Ti/TiO_2_ interference coatings. Appl. Surf. Sci..

[17] Skowronski L., Wachowiak A., Zdunek K., Trzcinski M., Naparty M. (2017). TiO_2_-based decorative coatings deposited on the AISI 316L stainless steel and glass using an industrial scale magnetron. Thin Solid Film..

[18] Zhang H., Banfield J.F. (1998). Thermodynamic analysis of phase stability of nanocrystalline titania. J. Mater. Chem..

[19] Zhang H., Banfield J.F. (2000). Understanding polymorphic phase transformation behavior during growth of nanocrystalline aggregates: Insights from TiO_2_. J. Phys. Chem. B.

[20] Nahar M.S., Zhang J., Hasegawa K., Kagaya S., Kuroda S. (2009). Phase transformation of anatase-rutile crystals in doped and undoped TiO_2_ particles obtained by the oxidation of polycrystalline sulfide. Mater. Sci. Semicond. Process..

[21] Hanaor D.A.H., Sorrell C.C. (2011). Review of the anatase to rutile phase transformation. J. Mater. Sci..

[22] Reidy D., Holmes J., Morris M. (2006). The critical size mechanism for the anatase to rutile transformation in TiO_2_ and doped-TiO_2_. J. Eur. Ceram. Soc..

[23] Choi H.C., Jung Y.M., Bin Kim S. (2005). Size effects in the Raman spectra of TiO_2_ nanoparticles. Vib. Spectrosc..

[24] Alsaiari M.A., Alhemiary N.A., Umar A., Hayden B.E. (2020). Growth of amorphous, anatase and rutile phase TiO_2_ thin films on Pt/TiO_2_/SiO_2_/Si (SSTOP) substrate for resistive random access memory (ReRAM) device application. Ceram. Int..

[25] Nezar S., Saoula N., Sali S., Faiz M., Mekki M., Laoufi N.A., Tabet N. (2017). Properties of TiO_2_ thin films deposited by rf reactive magnetron sputtering on biased substrates. Appl. Surf. Sci..

[26] Sutiono H., Tripathi A.M., Chen H.-M., Chen C.-H., Su W.-N., Chen L.-Y., Dai H., Hwang B.-J. (2016). Facile synthesis of [101]-oriented rutile TiO_2_ nanorod array on FTO substrate with a tunable anatase-rutile heterojunction for efficient solar water splitting. ACS Sustain. Chem. Eng..

[27] Juma A., Acik I.O., Mikli V., Mere A., Krunks M. (2015). Effect of solution composition on anatase to rutile transformation of sprayed TiO_2_ thin films. Thin Solid Film..

[28] Zhang Q., Li C. (2020). High temperature stable anatase phase titanium dioxide films synthesized by mist chemical vapor deposition. Nanomaterials.

[29] Ricci P.C., Carbonaro C.M., Stagi L., Salis M., Casu A., Enzo S., Delogu F. (2013). Anatase-to-rutile phase transition in TiO_2_ nanoparticles irradiated by visible light. J. Phys. Chem. C.

[30] Shannon R.D., Pask J.A. (1965). Kinetics of the anatase-rutile transformation. J. Am. Ceram. Soc..

[31] Mali M.G., Yoon H., An S., Choi J.-Y., Kim H.-Y., Lee B.C., Kim B.N., Park J.H., Al-Deyab S.S., Yoon S.S. (2014). Enhanced solar water splitting of electron beam irradiated titania photoanode by electrostatic spray deposition. Appl. Surf. Sci..

[32] Prasad K., Pinjari D., Pandit A., Mhaske S. (2010). Phase transformation of nanostructured titanium dioxide from anatase-to-rutile via. combined ultrasound assisted sol-gel technique. Ultrason. Sonochemistry.

[33] Tripathi A.K., Singh M.K., Mathpal M., Mishra S.K., Agarwal A. (2013). Study of structural transformation in TiO_2_ nanoparticles and its optical properties. J. Alloy. Compd..

[34] Skowronski L., Zdunek K., Nowakowska-Langier K., Chodun R., Trzcinski M., Kobierski M., Kustra M., Wachowiak A., Wachowiak W., Hiller T. (2015). Characterization of microstructural, mechanical and optical properties of TiO_2_ layers deposited by GIMS and PMS methods. Surf. Coat. Technol..

[35] Ciesielski A., Skowroński Ł., Trzcinski M., Gorecka E., Trautman P., Szoplik T. (2018). Evidence of germanium segregation in gold thin films. Surf. Sci..

[36] Ciesielski A., Skowroński Ł., Trzcinski M., Szoplik T. (2017). Controlling the optical parameters of self-assembled silver films with wetting layers and annealing. Appl. Surf. Sci..

[37] Ciesielski A., Trzcinski M., Szoplik T. (2020). Inhibiting the segregation of germanium in silver nanolayers. Crystals.

[38] Garlisi C., Scandura G., Szlachetko J., Ahmadi S., Sa J., Palmisano G. (2016). E-beam evaporated TiO_2_ and Cu-TiO_2_ on glass: Performance in the discoloration of methylene blue and 2-propanol oxidation. Appl. Catal. A Gen..

[39] Agarwal D.C., Chauhan R.S., Kumar A., Kabiraj D., Singh F., Khan S.A., Avasthi D.K., Pivin J.C., Kumar M., Ghatak J. (2006). Synthesis and characterization of ZnO thin film grown by electron beam evaporation. J. Appl. Phys..

[40] Al Asmar R., Zaouk D., Bahouth P., Podleki J., Foucaran A. (2006). Characterization of electron beam evaporated ZnO thin films and stacking ZnO fabricated by e-beam evaporation and rf magnetron sputtering for the realization of resonators. Microelectron. Eng..

[41] Skowronski L., Ciesielski A., Olszewska A., Szczesny R., Naparty M., Trzcinski M., Bukaluk A. (2020). Microstructure and optical properties of e-beam evaporated zinc oxide films—effects of decomposition and surface desorption. Materials.

[42] Biesinger M.C., Lau L.W.M., Gerson A.R., Smart R.S.C. (2010). Resolving surface chemical states in XPS analysis of first row transition metals, oxides and hydroxides: Sc, Ti, V, Cu and Zn. Appl. Surf. Sci..

[43] Mayer J., Diebold U., Madey T., Garfunkel E. (1995). Titanium and reduced titania overlayers on titanium dioxide (110). J. Electron Spectrosc. Relat. Phenom..

[44] Perron H., Vandenborre J., Domain C., Drot R., Roques J., Simoni E., Ehrhardt J.-J., Catalette H. (2007). Combined investigation of water sorption on TiO_2_ rutile (110) single crystal face: XPS vs. periodic DFT. Surf. Sci..

[45] Fengler S., Kriegel H., Schieda M., Gutzmann H., Thomas Klassen T., Wollgarten M., Dittrich T. (2020). Charge transfer in c-Si(n^++^)/TiO_2_(ALD) at the amorphous/anatase transition: A transient surface photovoltage spectroscopy study. ACS Appl. Mater. Interfaces.

[46] Rosenthal D., Zizak I., Darowski N., Magkoev T.T., Christmann K. (2006). The growth and structure of titanium dioxide films on a Re(1 0-1 0) surface: Rutile(0 1 1)-(2 × 1). Surf. Sci..

[47] Männig A., Zhao Z., Rosenthal D., Christmann K., Hoster H., Rauscher H., Behm R. (2005). Structure and growth of ultrathin titanium oxide films on Ru(0 0 0 1). Surf. Sci..

[48] Lai X., Guo Q., Min B., Goodman D. (2001). Synthesis and characterization of titania films on Mo(1 1 0). Surf. Sci..

[49] Mitchell D., Attard D., Triani G. (2003). Transmission electron microscopy studies of atomic layer deposition TiO_2_ films grown on silicon. Thin Solid Film..

[50] Woollam J.A. (2010). Guide to Using WVASE32^®^.

[51] Fujiwara H. (2009). Spectroscopic Ellipsometry. Principles and Applications.

[52] Skowroński Ł., Antończak A., Trzcinski M., Łazarek Ł., Hiller T., Bukaluk A., Wronkowska A. (2014). Optical properties of laser induced oxynitride films on titanium. Appl. Surf. Sci..

[53] Tauc J.T. (1974). Amorphous and Liquid Semiconductors.

[54] Nowakowska-Langier K., Skowronski L., Chodun R., Okrasa S., Strzelecki G.W., Wilczopolska M., Wicher B., Mirowski R., Zdunek K. (2020). Influence of generation control of the magnetron plasma on structure and properties of copper nitride layers. Thin Solid Film..

[55] Takikawa H., Matsui T., Sakakibara T., Bendavid A., Martin P.J. (1999). Properties of titanium oxide film prepared by reactive cathodic vacuum arc deposition. Thin Solid Film..

[56] Miao L., Jin P., Kaneko K., Terai A., Nabatova-Gabain N., Tanemura S. (2003). Preparation and characterization of polycrystalline anatase and rutile TiO_2_ thin films by rf magnetron sputtering. Appl. Surf. Sci..

[57] Ohno T., Numakura K., Itoh H., Suzuki H., Matsuda T. (2009). Control of the quantum size effect of TiO_2_-SiO_2_ hybrid particles. Mater. Lett..

[58] Bendavid A., Martin P., Takikawa H. (2000). Deposition and modification of titanium dioxide thin films by filtered arc deposition. Thin Solid Films.

